# Comparing Outcomes of Oligometastases Treated with Hypofractionated Image-Guided Radiotherapy (HIGRT) with a Simultaneous Integrated Boost (SIB) Technique versus Metastasis Alone: A Multi-Institutional Analysis

**DOI:** 10.3390/cancers14102403

**Published:** 2022-05-13

**Authors:** Rachel F. Shenker, Jeremy G. Price, Corbin D. Jacobs, Manisha Palta, Brian G. Czito, Yvonne M. Mowery, John P. Kirkpatrick, Matthew J. Boyer, Taofik Oyekunle, Donna Niedzwiecki, Haijun Song, Joseph K. Salama

**Affiliations:** 1 Department of Radiation Oncology, Duke University School of Medicine, Durham, NC 27710, USA; rachel.shenker@duke.edu (R.F.S.); jeremy.price@tuhs.temple.edu (J.G.P.); corbinjacobs@gmail.com (C.D.J.); manisha.palta@duke.edu (M.P.); brian.czito@duke.edu (B.G.C.); yvonne.mowery@duke.edu (Y.M.M.); john.kirkpatrick@duke.edu (J.P.K.); matthew.boyer@duke.edu (M.J.B.); haijun.song@duke.edu (H.S.); 2 Department of Radiation Oncology, Lewis Katz School of Medicine at Temple University, Philadelphia, PA 19140, USA; 3 Cancer Care Northwest, Coeur d’Alene, ID 83814, USA; 4 Department of Head and Neck Cancer & Communication Sciences, Duke University School of Medicine, Durham, NC 27710, USA; 5 Durham Veterans Affairs Health Care System, Radiation Oncology Service, Durham, NC 27705, USA; 6 Department of Biostatistics, Duke University, Durham, NC 27710, USA; oyekunle.toafik@duke.edu (T.O.); donna.niedzwiecki@duke.edu (D.N.)

**Keywords:** oligometastases, radiation, integrated boost

## Abstract

**Simple Summary:**

Hypofractionated image-guided radiotherapy (HIGRT) is a common method in which high doses of radiation are delivered to treat oligometastatic disease. We have previously reported on the clinical outcomes of treating oligometastases with radiation using an elective simultaneous integrated boost technique (SIB), delivering higher doses to known metastases and reduced doses to adjacent bone or nodal basins. Here we compare outcomes of oligometastases receiving radiation targeting metastases alone (MA) versus those treated via an SIB. Both SIB and MA irradiation of oligometastases achieved high rates of tumor metastases control and similar pain control. Further investigation of this technique with prospective trials is warranted.

**Abstract:**

Purpose: We previously reported on the clinical outcomes of treating oligometastases with radiation using an elective simultaneous integrated boost technique (SIB), delivering higher doses to known metastases and reduced doses to adjacent bone or nodal basins. Here we compare outcomes of oligometastases receiving radiation targeting metastases alone (MA) versus those treated via an SIB. Methods: Oligometastatic patients with ≤5 active metastases treated with either SIB or MA radiation at two institutions from 2013 to 2019 were analyzed retrospectively for treatment-related toxicity, pain control, and recurrence patterns. Tumor metastasis control (TMC) was defined as an absence of progression in the high dose planning target volume (PTV). Marginal recurrence (MR) was defined as recurrence outside the elective PTV but within the adjacent bone or nodal basin. Distant recurrence (DR) was defined as any recurrence that is not within the PTV or surrounding bone or nodal basin. The outcome rates were estimated using the Kaplan–Meier method and compared between the two techniques using the log-rank test. Results: 101 patients were treated via an SIB to 90 sites (58% nodal and 42% osseous) and via MA radiation to 46 sites (22% nodal and 78% osseous). The median follow-up among surviving patients was 24.6 months (range 1.4–71.0). Of the patients treated to MA, the doses ranged from 18 Gy in one fraction (22%) to 50 Gy in 10 fractions (50%). Most patients treated with an SIB received 50 Gy to the treated metastases and 30 Gy to the elective PTV in 10 fractions (88%). No acute grade ≥3 toxicities occurred in either cohort. Late grade ≥3 toxicity occurred in 3 SIB patients (vocal cord paralysis and two vertebral body compression), all related to the high dose PTV and not the elective volume. There was similar crude pain relief between cohorts. The MR-free survival rate at 2 years was 87% (95% CI: 70%, 95%) in the MA group and 98% (95% CI: 87%, 99%) in the SIB group (*p* = 0.07). The crude TMC was 89% (41/46) in the MA group and 94% (85/90) in the SIB group. There were no significant differences in DR-free survival (65% (95% CI: 55–74%; *p* = 0.24)), disease-free survival (60% (95% CI: 40–75%; *p* = 0.40)), or overall survival (88% (95% CI: 73–95%; *p* = 0.26)), between the MA and SIB cohorts. Conclusion: Both SIB and MA irradiation of oligometastases achieved high rates of TMC and similar pain control, with a trend towards improved MR-free survival for oligometastases treated with an SIB. Further investigation of this technique with prospective trials is warranted.

## 1. Introduction

Since the description of the clinical state of oligometastases (OM) in the 1990s [1], numerous randomized clinical trials have shown improved progression-free and overall survival (OS) outcomes with the delivery of metastasis-directed therapy to limited metastases, including resection [2,3] and ablative radiotherapy to metastatic sites of disease [4,5,6,7]. For patients with OM and poor performance status, large metastasis size, and/or numerous metastases in multiple organs, radiation therapy is often the preferred modality as it allows for the quick resumption of systemic therapy with minimal interruption and high rates of treated tumor control with an acceptable side effect profile. However, the optimal technique in which radiotherapy is delivered to OM is not well established.

Stereotactic body radiotherapy (SBRT), also called stereotactic ablative radiotherapy (SABR) and more precisely described as hypofractionated image-guided radiotherapy (HIGRT), is a common radiation technique used to treat OM, delivering high doses per fraction to limited target volumes [5,8,9]. However, following metastasis-directed radiation therapy (MDT), progression in nearby nodal basins or bones is common [10,11]. We previously reported clinical outcomes of OM treated with a simultaneous integrated boost technique (SIB), delivering higher doses to known metastases and reduced doses to adjacent bones/nodal basins [12]. This previous study demonstrated high rates of treated metastasis control (TMC), pain control, limited marginal progression, and acceptable toxicity. Here we aim to compare outcomes of patients with OM who received radiation targeting metastases alone (MA) versus those treated with an elective volume receiving a lower dose and gross tumor volume receiving a higher dose using a SIB. We hypothesized that the SIB technique would maintain TMC while reducing marginal recurrences (MRs).

## 2. Methods

### 2.1. Patient Selection

Methods, including patient selection and the treatment technique for patients treated with the SIB technique, have been previously described [12]. In brief, for all patients, those >18 years of age with pathologically confirmed solid tumor malignancy of any primary site and five or fewer active metastatic sites (including de novo, oligorecurrent, and oligoprogressive metastasis that have not yet been controlled with local therapy) treated with radiation to nodal and/or osseous metastases with either SIB or MA at Duke University Medical Center or the Durham Veterans’ Affairs Medical Center from 1 January 2013 through 1 January 2019 were identified and included in this analysis. The National Comprehensive Cancer Network staging imaging recommendations per primary tumor site were used to quantify the number of active metastases. This study was approved by both respective Institutional Review Boards (IRB) (Duke Health System IRB Pro00101071 and Durham VA Health Care System IRB 01740).

Relevant patient characteristics included age at the time of radiotherapy, sex, primary tumor site and histology, type of primary and metastasis directed treatment type, number of metastases, the volume of metastases, presence of painful metastases, date of birth, date of death, dates of last follow up, and time from diagnosis to metastatic disease. Further treatment characteristics included were the dose of radiation to both the primary and metastatic sites, the number of fractions per treatment, and the volume of gross tumor volume (GTV), and planning target volume (PTV). These data were collected via retrospective chart review.

### 2.2. Treatment Technique

**Figure 1:**
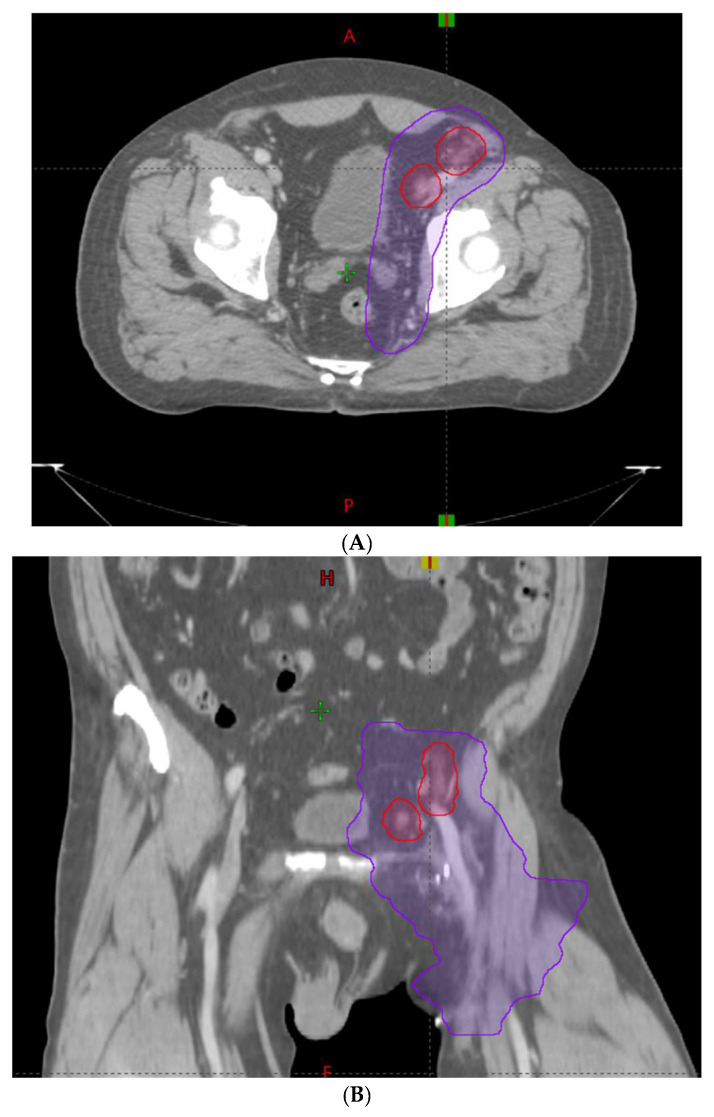
Treatment of oligometastatic external iliac lymph nodes with a simultaneous integrated boost (SIB) technique. Red line = Planning target volume (PTV) prescribed to 50 Gy. Purple line = PTV prescribed to 30 Gy. (**A**) = Axial view. (**B**) = Coronal view.

**Figure 2:**
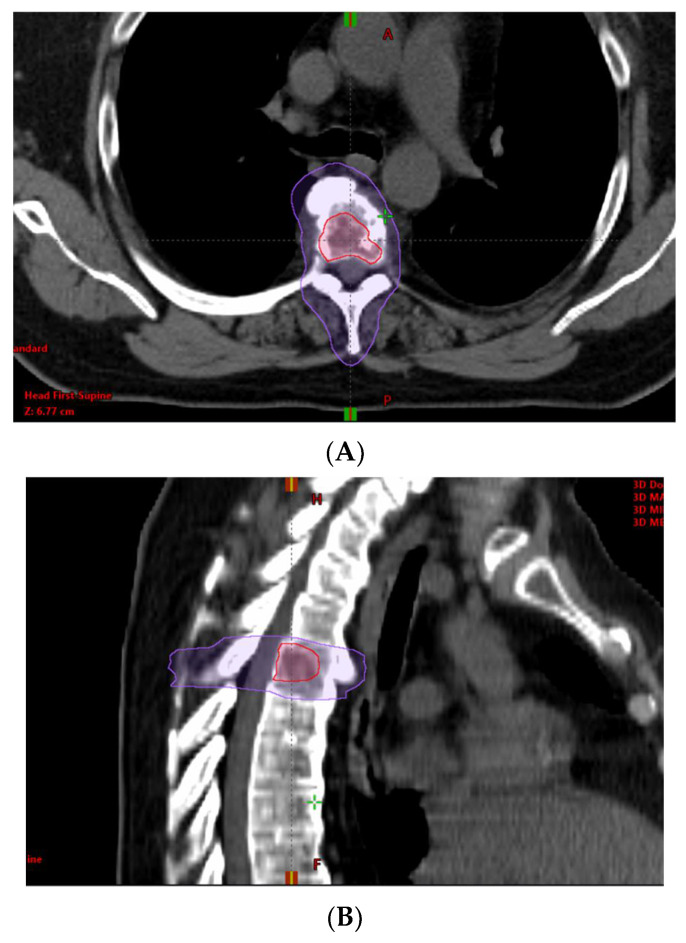
Treatment of oligometastatic T5 lesion with a simultaneous integrated boost (SIB) technique. Red line = Planning target volume (PTV) prescribed to 50 Gy. Purple line = PTV prescribed to 30 Gy. (**A**) = Axial view. (**B**) = Sagittal view.

Prior to initiating radiation treatment, treatment planning images were collected using a CT simulation. Patients were set up in a custom immobilization device with intravenous (IV) contrast as indicated. GTV was identified and contoured on each axial slice and expanded by 2–7 mm in each direction to develop a PTV (PTVboost) for MA-directed oligometastases. An elective CTV was contoured from the GTV for patients who received the SIB techniques. This CTV included the GTV and either the surrounding nodal chain or bone. This CTV was then expanded by 5–7 mm radially to develop an elective PTV (PTVelect). A boost PTV (PTVboost) was developed by expanding the GTV radially by 0–5 mm. Most oligometastases were treated with 50 Gy in 10 fractions to the PTVboost and 30 Gy in 10 fractions to the PTVelect. The typical planning targets are shown in Figure 1 and Figure 2.

The treatments involving the PTVboost for all of the patients were selectively underdosed to meet previously defined dose constraints for organs at risk [13,14,15]. Patients were treated via a linear accelerator with conformal radiation therapy, including volumetric modulated arc therapy (VMAT) or intensity-modulated radiotherapy (IMRT). Image guidance was routinely used for treatment, which typically included cone beam computed tomography (CBCT) and on-board imaging (OBI). During the treatment, patients were assessed weekly for toxicity. Follow-up clinic visits and imaging were performed at the discretion of the treating physician. Systemic therapy was managed at the discretion of the medical oncologist.

### 2.3. Outcomes

The overall survival (OS) was defined as the time from the start of the metastasis-directed radiation to death or the most recent follow-up. Disease-free survival (DFS) was defined as the time from the start of the metastasis-directed therapy to the first recorded recurrence or death. Marginal recurrence (MR) was defined as a recurrence within the same bone, organ, or lymph node basin as the treated metastasis. Distant recurrence (DR) was defined as a recurrence outside of the PTV, same bone, organ, or lymph node. A metastatic recurrence included any recurrence within the treated PTV. This was measured by tumor metastasis control (TMC). The primary outcome of this study was both TMC and MR. Five independent authors (RS, JP, CJ, JS, and MM) reviewed all of the eligible cases, and a group consensus was reached for each individually treated metastasis.

Acute and late toxicities were recorded via the Common Terminology Criteria for Adverse Events (CTCAE) version 5.0. The presence and resolution of pain for a treated metastatic lesion was included. Freedom from pain recurrence (FFPR) was determined by the time from the metastasis-directed therapy to the return of pain (if it occurred).

### 2.4. Statistical Analysis

Clinical endpoints, including OS, DFS, MR, and DR, were estimated using the Kaplan–Meier method. The difference in TMC and clinical endpoints were compared between groups using a log-rank test. Statistical analysis was performed using R version 3.4.3.

Patient and treatment characteristics, as previously specified, were summarized with median and interquartile ranges (IQR) for continuous variables. Median time to follow-up was calculated for all patients from the start of metastasis-directed therapy until the date of death or the last recorded follow-up. Rates of DR, OS, and DFS were calculated on a per patient analysis. MR and FFPR were calculated on a per metastasis analysis.

## 3. Results

### 3.1. Baseline Characteristics

Between July 2013 and January 2019, a total of 101 patients were treated using either the SIB technique at 90 sites (53% nodal and 47% osseous) or MA radiation at 46 sites (13% nodal and 87% osseous). There were 108 discrete radiotherapy courses, 68 treated with SIB and 40 treated with MA (some patients were treated more than once). Demographic, disease, and treatment characteristics are summarized in Table 1. The most common primary tumors were prostate (37%), lung (15%), and breast (7%) among all patients. The median time from diagnosis to first metastasis was 31 months for all patients. The median ages for SIB and MA radiation were 69 and 66 years, respectively. The median follow-up among surviving patients was 24.6 months (range 1.4–71.0) for all patients.

The majority of the patients in both groups had one metastatic lesion treated (43% in SIB and 59% in MA). Approximately half (51%) of the OM treated with SIB were located in the abdominopelvic region, whereas the most common site treated with MA radiation was the spine (41%). Within the MA cohort, prescribed doses ranged from 18 Gy in 1 fraction (22%) to 50 Gy in 10 fractions (50%). Most patients treated with SIB received 50 Gy to the treated metastases and 30 Gy to the elective PTV in 10 fractions (88%). The median GTV volume was 11.6 cm^3^ in the SIB group and 5.6 cm^3^ in the MA group. The median high dose PTV for SIB (PTVboost) and MA cohorts were 28.3 cm^3^ and 34.3 cm^3^, respectively. The median PTVelect in the SIB cohort was 229.2 cm^3^.

### 3.2. Toxicity and Pain Analysis

The toxicity for all patients is summarized in Table 2. A grade 1–2 toxicity was recorded for 48% of all treatment courses. The toxicity profile for the MA cohort is summarized in Table 3. One patient was noted to have an acute grade 3 toxicity that correlated with recent chemotherapy (docetaxel) administration. Therefore, this toxicity was likely related to systemic therapy. Toxicity profile for the SIB cohort is summarized in Table 4. Late grade ≥3 toxicity occurred in 3 SIB patients (vocal cord paralysis n = 2, vertebral body compression n = 2) and no MA patients. For the SIB patient with late vocal cord paralysis, the vocal cord was in the PTVboost. Therefore, it is likely that this toxicity would have occurred in either HIGRT-MA or HIGRT-SIB technique. An additional patient in the SIB cohort had a recorded late grade 3 vocal cord paralysis that was noted prior to starting HIGRT. Both patients that experienced vertebral body compression had lytic lesions with lytic components 50% of the height of the vertebral body. Therefore, it is likely that this toxicity may have resulted from either treatment technique. There was similar crude pain relief between cohorts: 82% with MA (9/11 patients reporting improved pain) and 86% with SIB (19/22).

### 3.3. Patterns of Recurrence

**Figure 3:**
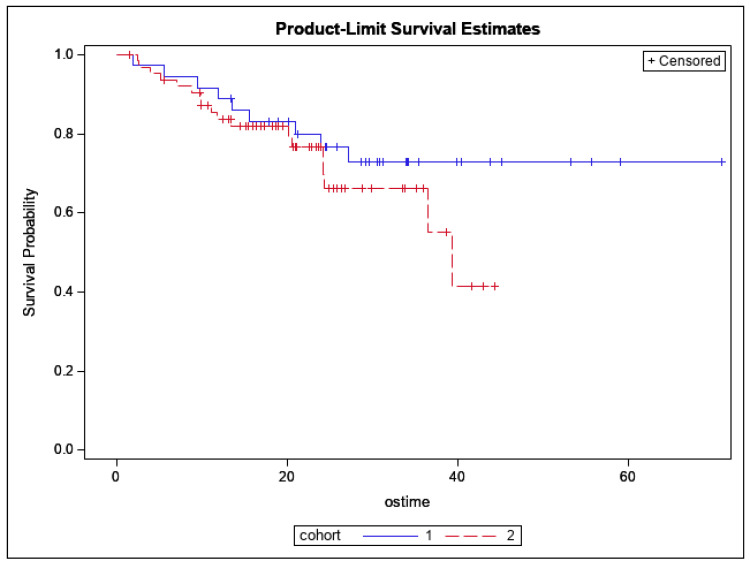
Kaplan–Meier curves depicting Overall Survival by Treatment Cohort for all patients (Blue = HIGRT Alone; Red = HIGRT + SIB; log rank *p* = 0.26).

**Figure 4:**
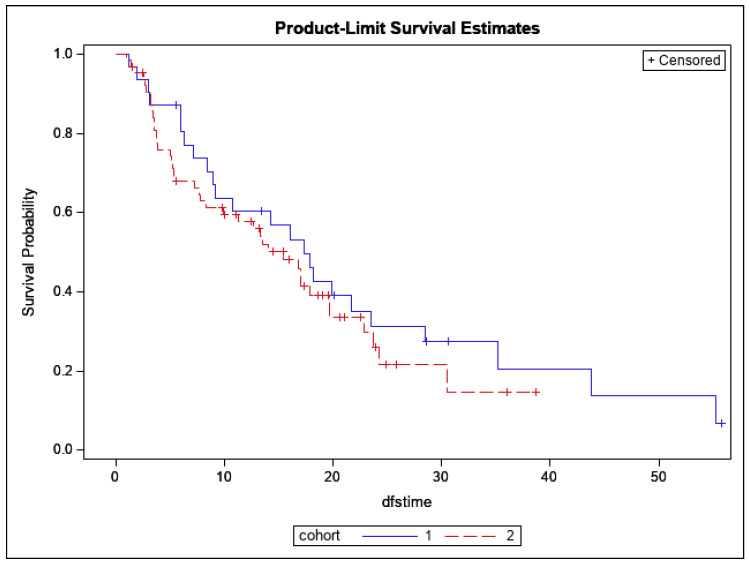
Kaplan–Meier curves depicting Disease-free Survival by Treatment Cohort (Blue = HIGRT Alone; Red = HIGRT + SIB); log rank *p* = 0.40.

**Figure 5:**
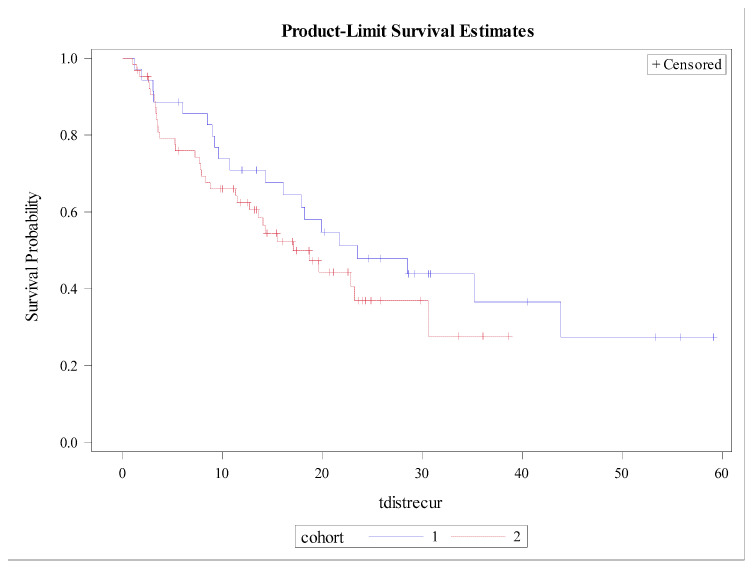
Kaplan–Meier curves depicting Distant Recurrence-free Survival by Treatment Cohort (Blue = HIGRT Alone; Red = HIGRT + SIB); log rank *p* = 0.24.

**Figure 6:**
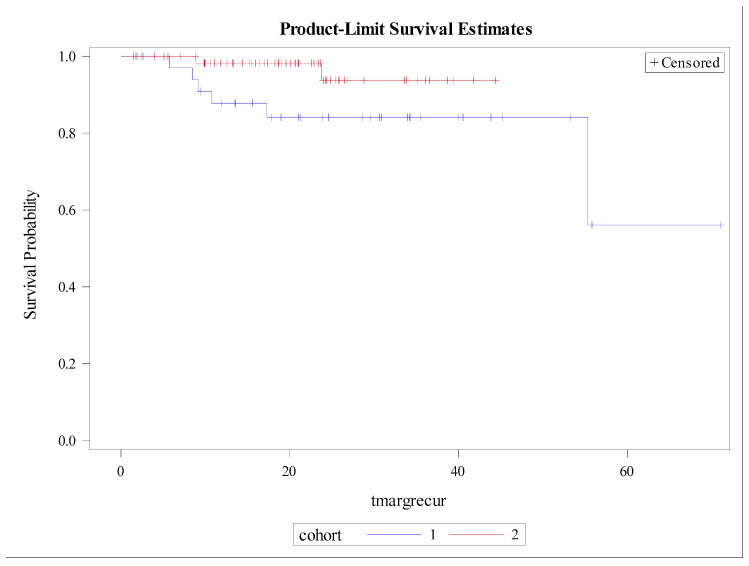
Kaplan–Meier curves depicting Marginal Recurrence-free Survival by Treatment Cohort (Blue = HIGRT Alone; Red = HIGRT + SIB); log rank *p* = 0.07.

There was no significant difference in 12-month OS, which was 88% (95% CI: 73–95%; *p* = 0.26), as shown in Figure 3. The 12-month DFS was 60% (95% CI: 40–75%; *p* = 0.40), as shown in Figure 4. There were no significant differences found between both cohorts in DR-free survival at 12 months, which was 65% (95% CI: 55–74%; *p* = 0.24) (Figure 5). The number of MR events was small (n = 8). The crude MR rates were more frequent in the MA group, 13% (n = 6), compared to the SIB group, 2% (n = 2). MR-free survival at 2 years was 87% (95% CI: 70–95%) in the MA group and 98% (95% CI: 87–99%) in the SIB group (*p* = 0.07) (Figure 6). The crude TMC was 89% (41/46) in the MA group and 94% (85/90) in the SIB group.

## 4. Discussion

Treatment of OM with ablative radiotherapy has been shown to be effective in controlling the progression of disease, but MR near the treated region remains common. In a previous report, we found that the addition of an elective, lower dose volume in addition to a high dose to the gross metastasis delivered with a SIB technique resulted in excellent TMC, rare MR, and was well tolerated with few toxicities [12]. In this expanded analysis, we compared outcomes of patients treated with an elective SIB approach to those only treated for gross disease only and confirmed a numerically reduced rate of MR with equivalent rates of TMC and palliation. Additionally, while more late side effects were seen in the SIB group, these were all related to the high dose treatment area and not related to the incorporation of the low dose elective boost. Therefore, similar toxicity would be expected if these patients had been treated with MA radiation with similar high dose PTV.

Perhaps most importantly, we saw a high rate of TMC in patients treated only for the metastases as well as for an elective volume. Compared to historical controls reporting the risk of MR in adjacent lymph nodal basins and structures [10,11,16,17,18,19,20,21], both cohorts had relatively small crude MR. Although this metric did not reach statistical significance when comparing both groups, there was a trend toward improved MR- free survival in the SIB group. This could theoretically decrease the risk of overlapping treatment sites if a new metastatic lesion were to develop nearby. There were no significant differences in OS, DFS, or DR, implicating that an HIGRT-SIB technique is an effective method that mirrors historical controls.

Both the SIB and MA techniques were well tolerated, with generally mild acute and late toxicities. No patient in either cohort experienced a grade 3 or worse acute toxicity related to radiotherapy. One patient was hospitalized for acute grade 3 lymphopenia, attributed to a recent dose of chemotherapy (docetaxel) [22]. The patient had also completed radiotherapy without issue and previously developed lymphopenia shortly after other chemotherapy cycles. There were three patients who experienced a late grade 3 toxicity: two experienced vertebral body compression, and one experienced vocal cord paralysis. Upon review of the patient record, it was noted that there had been concern that vocal cord paralysis was likely due to high dose radiation. However, it was noted to be within the high dose treatment volume and would have likely resulted with either technique. In the recently reported NRG BR-001 phase I trial evaluating the safety of SBRT in patients with multiple metastases, 17% of patients experienced a late grade 3 toxicity that was deemed due to radiotherapy [23]. It is of note that in this trial, multiple sites were treated at once, and many patients within our study had one site treated at a given time. In the phase II SABR-COMET trial, 29% of patients experienced grade 2 or more toxicities following SBRT [7]. Overall, patients in both cohorts had excellent toxicity profiles in comparison to other published reports [24]. Our data also mirrors other studies that have previously reported acceptable toxicity profiles in the setting of HIGRT in oligometastatic disease [25,26,27,28].

The addition of systemic therapy was noted to be given at the discretion of our partnering medical oncologists before, during, and after the treatment of oligometastases. The role of systemic therapy in the setting of local treatment of oligometastases is an evolving source of research interest. In a phase II trial by Gomez et al., patients with oligometastatic NSCLC were treated with first-line systemic therapy, followed by consolidative local therapy experienced a significant PFS benefit (11.9 months vs 3.9 months with systemic therapy alone) [4]. Of note, systemic therapy was not given concurrently with local therapy. In the setting of immunotherapy, there is interest in an abscopal phenomenon, in which an immune-mediated response following ablative therapy to one metastatic site may cause a response in other metastatic sites [29]. As our treatment strategies, techniques, and artificial intelligence continue to develop, personalized treatment plans per oligometastases may be possible [30].

There are several limitations to our retrospective study. Due to the nature of a retrospective study, it is subject to the limits of data that can be abstracted from medical records. It is also subject to the effects of possible confounding factors that would not be identified on an individual basis. In this study specifically, there are OM from multiple primary disease types treated, which make it difficult to discern which patients may benefit more from an SIB or MA technique. Although prospective studies such as SABR-COMET included multiple primary disease sites, the inherent biology of different tumors may affect the outcomes based on each radiotherapy technique. There are several randomized trials that are currently investigating the role of local treatment in site-specific oligometastases, including the VA STARPORT trial (NCT04787744) for prostate cancer and NRG-BR002 (NCT02364557) for breast cancer. It would be important for future studies comparing radiation techniques to have proper randomization and sufficient power for each disease site. Additionally, there were endpoints with rare events that precluded further analysis regarding pain control. While there was a trend towards improved MR-free survival in the SIB cohort, this study is not adequately powered to detect a significant difference. It is of note in our study that the dosing is different between both groups. While this is inherent due to the treatment technique, it is possible that similar sites and primary histologies between both groups may be treated with either more or less of a dose. The ideal dose for HIGRT to oligometastasis has not been clearly established, and therefore, dosing was chosen in our study based on previous studies [5,12,23,28,31], location, size, and feasibility to reach normal tissue constraints. Lastly, as a non-randomized, observational study, there is inherent selection bias by multiple providers in the choice of MA or SIB-HIGRT.

## 5. Conclusions

In conclusion, SIB-HIGRT with an elective lower dose PTV, as well as HIGRT to the metastasis alone, are both reasonable, safe options for patients with oligometastatic disease. Both SIB and MA HIGRT of OM achieved high rates of TMC and similar pain control, with a trend towards improved MR-free survival for OM treated with a SIB. Although more late grade 3 toxicities were seen in the SIB cohort, these were mechanistically related to the high-dose PTV and not the elective volume. Further investigation of this technique with prospective trials is warranted.

## Figures and Tables

**Table 1:** Patient and tumor characteristics.

	HIGRT-SIB	HIGRT-MA
Age, Median (range)	69	66
Gender, n (%)		
Female	10 (10%)	20 (20%)
Male	52 (51%)	19 (19%)
Primary Tumor Site, n (%)		
Prostate	36 (40%)	14 (30%)
Breast	0 (0%)	9 (20%)
Lung	15 (17%)	6 (13%)
Gastrointestinal	14 (16%)	3 (7%)
Kidney	2 (2%)	6 (13%)
Thyroid	3 (3%)	3 (7%)
Skin	3 (3%)	0 (0%)
Head and Neck	1 (1%)	0 (0%)
Testicle	1 (1%)	0 (0%)
Gynecologic	1 (1%)	0 (0%)
Other	14 (16%)	5 (10%)
Months from diagnosis to first metastasis, median (IQR)	44.75 (IQR: 11.9–73.1)	55.1 (IQR: 0–55.1)
Number of active metastases at the time of HIGRT, n (%)		
1	39 (43%)	27 (59%)
2	35 (39%)	13 (28%)
3	7 (8%)	6 (13%)
4	1 (1%)	0 (0%)
5	8 (9%)	0 (0%)
TREATED METASTASIS-SPECIFIC VARIABLE		
HIGRT target, n (%)		
Lymph node metastasis	48 (53%)	6 (13%)
Painful osseous metastasis	22 (24%)	11 (24%)
Non-painful osseous metastasis	20 (23%)	29 (63%)
HIGRT anatomic location, n (%)		
Abdominopelvic	46 (51%)	12 (26%)
Spine	16 (18%)	19 (41%)
Sternum or rib	11 (12%)	6 (13%)
Supraclavicular fossa, mediastinum, or axilla	16 (18%)	7 (15%)
Extremity	1 (1%)	2 (5%)
Greatest diameter of largest metastasis in cm, Median, (IQR)	2.6 (1.7–3.6)	2.7 (1.8–3.3)
GTV in cm^3^, Median (IQR)	11.6 (3.9–20.6)	5.6 (2.3–17.3)
PTVboost in cm^3^, Median (IQR)	28.3 (11.9–54.2)	34.3 (18.7–73.9)
PTVelect in cm^3^, Median (IQR)	229.2 (111.6–346.4)	N/A
Dose to PTVelect in Gy, Median (IQR)	30 (30–30)	N/A
Dose to PTVboost in Gy, Median (IQR)	50 (50–50)	30 (18–50)
HIGRT Fractions, Median (IQR)	10 (10–10)	7 (1–10)
HIGRT duration in days, Median (IQR)	13 (11–14)	10.5 (0–13)

**Table 2:** Toxicity profile for all courses (N = 108).

All Patients Toxicity	Acute Grade 1–2 N (%)	Acute Grade ≥ 3 N (%)	Late Grade 1–2 N (%)	Late Grade ≥ 3 N (%)
GI ^1^	31 (29)	0 (0)	3 (2)	0 (0)
GU ^2^	2 (2)	0 (0)	0 (0)	0 (0)
Hematologic	2 (2)	1 (1)	0 (0)	0 (0)
Neurologic	5 (5)	0 (0)	3 (2)	4 (3)
Respiratory	2 (2)	0 (0)	0 (0)	0 (0)
General	52 (48)	0 (0)	4 (4)	0 (0)
Pain Flare	5 (5)	0 (0)		

^1^ Gastrointestinal; ^2^ Genitourinary.

**Table 3:** Toxicity profile for HIGRT-MA per treatment course (N = 40).

Toxicity	Acute Grade 1–2 N (%)	Acute Grade ≥ 3 N (%)	Late Grade 1–2 N (%)	Late Grade ≥ 3 N (%)
GI ^1^	4 (10)	0 (0)	0 (0)	0 (0)
GU ^2^	0 (0)	0 (0)	0 (0)	0 (0)
Hematologic	0 (0)	0 (0)	0 (0)	0 (0)
Neurologic	1 (3)	0 (0)	0 (0)	0 (0)
Respiratory	0 (0)	0 (0)	0 (0)	0 (0)
General	9 (23)	0 (0)	1 (3)	0 (0)
Pain Flare	0 (0)	0 (0)		

^1^ Gastrointestinal; ^2^ Genitourinary.

**Table 4:** Toxicity profile for HIGRT + SIB per treatment course (N = 68).

Toxicity	Acute Grade 1–2 N (%)	Acute Grade ≥ 3 N (%)	Late Grade 1–2 N (%)	Late Grade ≥ 3 N (%)
GI ^1^	27 (40)	0 (0)	3 (4)	0 (0)
GU ^2^	2 (3)	0 (0)	0 (0)	0 (0)
Hematologic	2 (3)	1 (1)	0 (0)	0 (0)
Neurologic	4 (6)	0 (0)	3 (4)	4 (6)
Respiratory	2 (3)	0 (0)	0 (0)	0 (0)
General	43 (63)	0 (0)	3 (4)	0 (0)
Pain Flare	5 (7)	0 (0)		

^1^ Gastrointestinal; ^2^ Genitourinary.

## Data Availability

Data are available upon request from authors and IRB approval.

## References

[1] Hellman S., Weichselbaum R.R. (1995). Oligometastases. J. Clin. Oncol..

[2] Pastorino U., Buyse M., Friedel G., Ginsberg R.J., Girard P., Goldstraw P., Johnston M., McCormack P., Pass H., Putnam J.B. (1997). Long-term results of lung metastasectomy: Prognostic analyses based on 5206 cases. J. Thorac. Cardiovasc. Surg..

[3] Fong Y., Cohen A.M., Fortner J.G., Enker W.E., Turnbull A.D., Coit D.G., Marrero A.M., Prasad M., Blumgart L.H., Brennan M.F. (1997). Liver resection for colorectal metastases. J. Clin. Oncol..

[4] Gomez D.R., Blumenschein G.R., Lee J.J., Hernandez M., Ye R., Camidge D.R., Doebele R.C., Skoulidis F., Gaspar L.E., Gibbons D.L. (2016). Local consolidative therapy versus maintenance therapy or observation for patients with oligometastatic non-small-cell lung cancer without progression after first-line systemic therapy: A multicentre, randomised, controlled, phase 2 study. Lancet Oncol..

[5] Palma D.A., Olson R., Harrow S., Gaede S., Louie A.V., Haasbeek C., Mulroy L., Lock M., Rodrigues G.B., Yaremko B.P. (2019). Stereotactic ablative radiotherapy versus standard of care palliative treatment in patients with oligometastatic cancers (SABR-COMET): A randomised, phase 2, open-label trial. Lancet.

[6] Iyengar P., Wardak Z., Gerber D.E., Tumati V., Ahn C., Hughes R.S., Dowell J.E., Cheedella N., Nedzi L., Westover K.D. (2018). Consolidative Radiotherapy for Limited Metastatic Non-Small-Cell Lung Cancer: A Phase 2 Randomized Clinical Trial. JAMA Oncol..

[7] Palma D.A., Olson R., Harrow S., Gaede S., Louie A.V., Haasbeek C., Mulroy L., Lock M., Rodrigues G.B., Yaremko B.P. (2020). Stereotactic Ablative Radiotherapy for the Comprehensive Treatment of Oligometastatic Cancers: Long-Term Results of the SABR-COMET Phase II Randomized Trial. J. Clin. Oncol..

[8] Al-Hallaq H.A., Chmura S., Salama J.K., Winter K.A., Robinson C.G., Pisansky T.M., Borges V., Lowenstein J.R., McNulty S., Galvin J.M. (2016). Rationale of technical requirements for NRG-BR001: The first NCI-sponsored trial of SBRT for the treatment of multiple metastases. Pract. Radiat. Oncol..

[9] Salama J.K., Milano M.T. (2014). Radical irradiation of extracranial oligometastases. J. Clin. Oncol..

[10] Chang E.L., Shiu A.S., Mendel E., Mathews L.A., Mahajan A., Allen P.K., Weinberg J.S., Brown B.W., Wang X.S., Woo S.Y. (2007). Phase I/II study of stereotactic body radiotherapy for spinal metastasis and its pattern of failure. J. Neurosurg. Spine.

[11] Koyfman S.A., Djemil T., Burdick M.J., Woody N., Balagamwala E.H., Reddy C.A., Angelov L., Suh J.H., Chao S.T. (2012). Marginal recurrence requiring salvage radiotherapy after stereotactic body radiotherapy for spinal metastases. Int. J. Radiat. Oncol. Biol. Phys..

[12] Jacobs C.D., Palta M., Williamson H., Price J.G., Czito B.G., Salama J.K., Moravan M.J. (2019). Hypofractionated Image-Guided Radiation Therapy with Simultaneous-Integrated Boost Technique for Limited Metastases: A Multi-Institutional Analysis. Front. Oncol..

[13] Milano M.T., Katz A.W., Schell M.C., Philip A., Okunieff P. (2008). Descriptive analysis of oligometastatic lesions treated with curative-intent stereotactic body radiotherapy. Int. J. Radiat. Oncol. Biol. Phys..

[14] Li Q., Swanick C.W., Allen P.K., Gomez D.R., Welsh J.W., Liao Z., Balter P.A., Chang J.Y. (2014). Stereotactic ablative radiotherapy (SABR) using 70 Gy in 10 fractions for non-small cell lung cancer: Exploration of clinical indications. Radiother. Oncol..

[15] Chance W.W., Nguyen Q.N., Mehran R., Welsh J.W., Gomez D.R., Balter P., Komaki R., Liao Z., Chang J.Y. (2017). Stereotactic ablative radiotherapy for adrenal gland metastases: Factors influencing outcomes, patterns of failure, and dosimetric thresholds for toxicity. Pract. Radiat. Oncol..

[16] Ost P., Jereczek-Fossa B.A., Van As N., Zilli T., Tree A., Henderson D., Orecchia R., Casamassima F., Surgo A., Miralbell R. (2016). Pattern of Progression after Stereotactic Body Radiotherapy for Oligometastatic Prostate Cancer Nodal Recurrences. Clin. Oncol..

[17] Decaestecker K., De Meerleer G., Lambert B., Delrue L., Fonteyne V., Claeys T., De Vos F., Huysse W., Hautekiet A., Maes G. (2014). Repeated stereotactic body radiotherapy for oligometastatic prostate cancer recurrence. Radiat. Oncol..

[18] Nguyen Q.N., Shiu A.S., Rhines L.D., Wang H., Allen P.K., Wang X.S., Chang E.L. (2010). Management of spinal metastases from renal cell carcinoma using stereotactic body radiotherapy. Int. J. Radiat. Oncol. Biol. Phys..

[19] Gerszten P.C., Burton S.A., Ozhasoglu C., Welch W.C. (2007). Radiosurgery for spinal metastases: Clinical experience in 500 cases from a single institution. Spine.

[20] Sogono P., Bressel M., David S., Shaw M., Chander S., Chu J., Plumridge N., Byrne K., Hardcastle N., Kron T. (2021). Safety, Efficacy, and Patterns of Failure After Single-Fraction Stereotactic Body Radiation Therapy (SBRT) for Oligometastases. Int. J. Radiat. Oncol. Biol. Phys..

[21] Chi A., Liao Z., Nguyen N.P., Xu J., Stea B., Komaki R. (2010). Systemic review of the patterns of failure following stereotactic body radiation therapy in early-stage non-small-cell lung cancer: Clinical implications. Radiother. Oncol..

[22] Kotsakis A., Sarra E., Peraki M., Koukourakis M., Apostolaki S., Souglakos J., Mavromanomakis E., Vlachonikolis J., Georgoulias V. (2000). Docetaxel-induced lymphopenia in patients with solid tumors: A prospective phenotypic analysis. Cancer.

[23] Chmura S., Winter K.A., Robinson C., Pisansky T.M., Borges V., Al-Hallaq H., Matuszak M., Park S.S., Yi S., Hasan Y. (2021). Evaluation of Safety of Stereotactic Body Radiotherapy for the Treatment of Patients with Multiple Metastases: Findings from the NRG-BR001 Phase 1 Trial. JAMA Oncol..

[24] Poon I., Erler D., Dagan R., Redmond K.J., Foote M., Badellino S., Biswas T., Louie A.V., Lee Y., Atenafu E.G. (2020). Evaluation of Definitive Stereotactic Body Radiotherapy and Outcomes in Adults with Extracranial Oligometastasis. JAMA Netw. Open.

[25] Fumagalli I., Bibault J.E., Dewas S., Kramar A., Mirabel X., Prevost B., Lacornerie T., Jerraya H., Lartigau E. (2012). A single-institution study of stereotactic body radiotherapy for patients with unresectable visceral pulmonary or hepatic oligometastases. Radiat. Oncol..

[26] Rusthoven K.E., Kavanagh B.D., Burri S.H., Chen C., Cardenes H., Chidel M.A., Pugh T.J., Kane M., Gaspar L.E., Schefter T.E. (2009). Multi-institutional phase I/II trial of stereotactic body radiation therapy for lung metastases. J. Clin. Oncol..

[27] Rusthoven K.E., Kavanagh B.D., Cardenes H., Stieber V.W., Burri S.H., Feigenberg S.J., Chidel M.A., Pugh T.J., Franklin W., Kane M. (2009). Multi-institutional phase I/II trial of stereotactic body radiation therapy for liver metastases. J. Clin. Oncol..

[28] Phillips R., Shi W.Y., Deek M., Radwan N., Lim S.J., Antonarakis E.S., Rowe S.P., Ross A.E., Gorin M.A., Deville C. (2020). Outcomes of Observation vs Stereotactic Ablative Radiation for Oligometastatic Prostate Cancer: The ORIOLE Phase 2 Randomized Clinical Trial. JAMA Oncol..

[29] Beckham T.H., Yang T.J., Gomez D., Tsai C.J. (2021). Metastasis-directed therapy for oligometastasis and beyond. Br. J. Cancer.

[30] Fionda B., Iezzi R., Tagliaferri L. (2021). Evolutionary game theory and oligometastatic patient: Considering the role of interventional oncology. Eur. Rev. Med. Pharmacol. Sci..

[31] Lievens Y., Guckenberger M., Gomez D., Hoyer M., Iyengar P., Kindts I., Romero A.M., Nevens D., Palma D., Park C. (2020). Defining oligometastatic disease from a radiation oncology perspective: An ESTRO-ASTRO consensus document. Radiother. Oncol..

